# Preoperative prediction by artificial intelligence for mastoid extension in pars flaccida cholesteatoma using temporal bone high-resolution computed tomography: A retrospective study

**DOI:** 10.1371/journal.pone.0273915

**Published:** 2022-10-03

**Authors:** Masahiro Takahashi, Katsuhiko Noda, Kaname Yoshida, Keisuke Tsuchida, Ryosuke Yui, Takara Nakazawa, Sho Kurihara, Akira Baba, Masaomi Motegi, Kazuhisa Yamamoto, Yutaka Yamamoto, Hiroya Ojiri, Hiromi Kojima

**Affiliations:** 1 Department of Otorhinolaryngology, Jikei University School of Medicine, Tokyo, Japan; 2 SIOS Technology Inc., Tokyo, Japan; 3 Department of Radiology, The Jikei University School of Medicine, Tokyo, Japan; Universidade Federal de Sao Paulo/Escola Paulista de Medicina (Unifesp/epm), BRAZIL

## Abstract

Cholesteatoma is a progressive middle ear disease that can only be treated surgically but with a high recurrence rate. Depending on the extent of the disease, a surgical approach, such as microsurgery with a retroarticular incision or transcanal endoscopic surgery, is performed. However, the current examination cannot sufficiently predict the progression before surgery, and changes in approach may be made during the surgery. Large amounts of data are typically required to train deep neural network models; however, the prevalence of cholesteatomas is low (1-in-25, 000). Developing analysis methods that improve the accuracy with such a small number of samples is an important issue for medical artificial intelligence (AI) research. This paper presents an AI-based system to automatically detect mastoid extensions using CT. This retrospective study included 164 patients (80 with mastoid extension and 84 without mastoid extension) who underwent surgery. This study adopted a relatively lightweight neural network model called MobileNetV2 to learn and predict the CT images of 164 patients. The training was performed with eight divided groups for cross-validation and was performed 24 times with each of the eight groups to verify accuracy fluctuations caused by randomly augmented learning. An evaluation was performed by each of the 24 single-trained models, and 24 sets of ensemble predictions with 23 models for 100% original size images and 400% zoomed images. Fifteen otolaryngologists diagnosed the images and compared the results. The average accuracy of predicting 400% zoomed images using ensemble prediction model was 81.14% (sensitivity = 84.95%, specificity = 77.33%). The average accuracy of the otolaryngologists was 73.41% (sensitivity, 83.17%; specificity, 64.13%), which was not affected by their clinical experiences. Noteworthily, despite the small number of cases, we were able to create a highly accurate AI. These findings represent an important first step in the automatic diagnosis of the cholesteatoma extension.

## Introduction

Cholesteatomas are benign collections of keratinized squamous epithelium mostly found within the middle ear; however, they are intractable chronic proliferative diseases that can cause fatal complications such as bone destruction and brain abscesses. The only treatment option is surgery, and even with appropriate surgery, the long-term recurrence rate is reported to be around 20–30% [1]. The various surgical procedures depend on the extent of the lesion, especially its extension to the mastoid. This affects surgical approaches, including the choice of minimally invasive endoscopic surgery or microsurgery with a postauricular incision [2]. However, the present imaging examinations cannot adequately confirm the extent of the lesion, which may necessitate intraoperative modification of the approach and significantly prolong the operative time [3].

**Fig 1:**
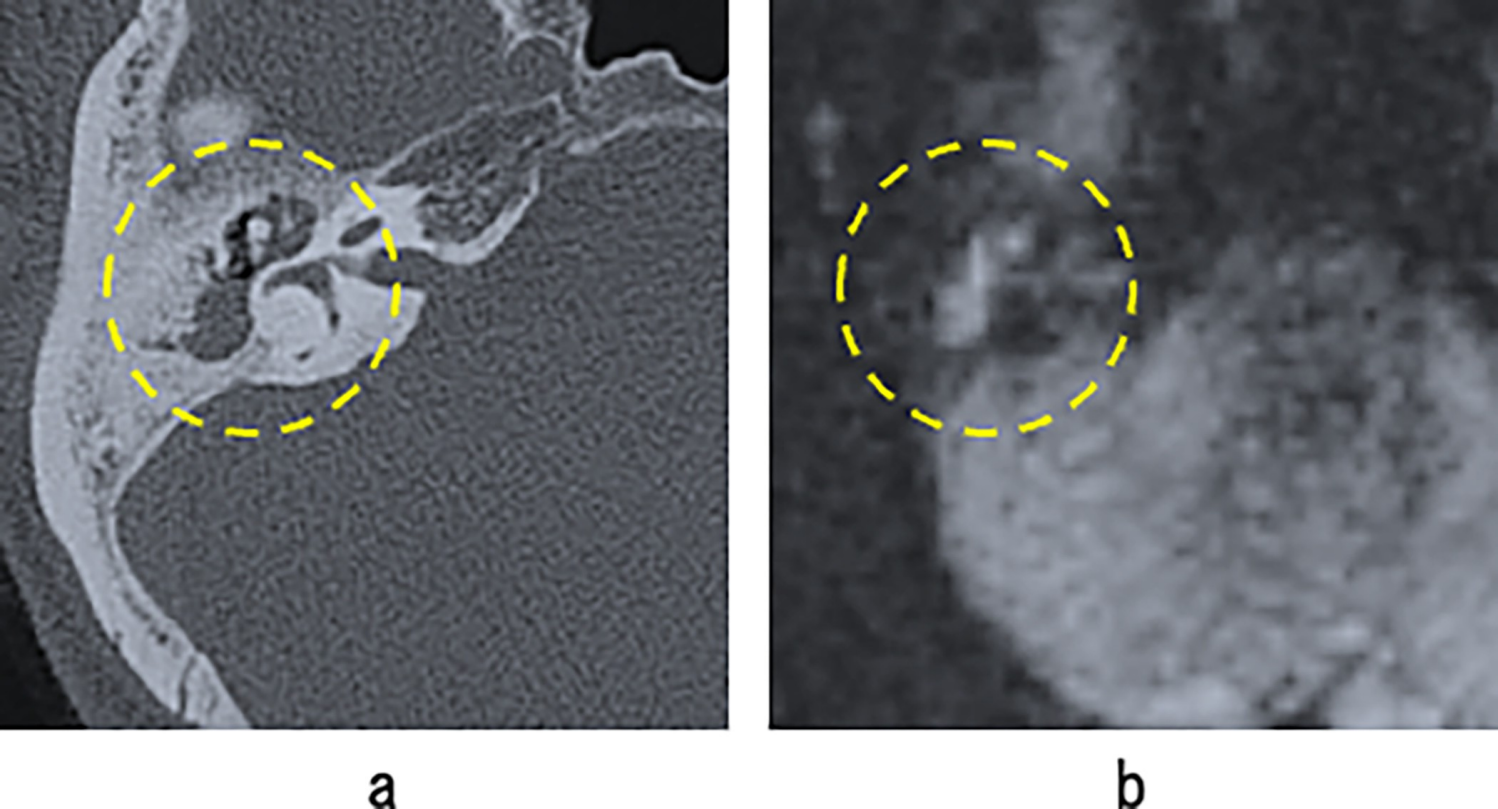
CT and MRI (non-EP diffusion-weighted imaging) findings in patients with cholesteatoma. Yellow dotted lines indicate lesions. (a) CT showing a soft density lesion from the attic to the mastoid. It is difficult to distinguish between cholesteatoma and non-cholesteatoma inflammatory lesions. (b) MRI can diagnose the condition but the exact extent is difficult to determine.

High-resolution computed tomography (HRCT) is useful in the evaluation of temporal bone lesions because it provides a direct view of the interior of the temporal bone and shows finer structural details than conventional computed tomography (CT). Therefore, in clinical practice, HRCT images are used for preoperative evaluation of the extent of cholesteatoma and its complications. It is difficult to distinguish between cholesteatoma and non-cholesteatoma inflammatory areas on CT because they show the similar densities (Fig 1A). An expanded aditus ad antrum on radiological imaging is indicative of mastoid extension of cholesteatoma [4]. However, there are no imaging criteria for the expansion of the aditus ad antrum; therefore, evaluation by radiologists or otolaryngologists lacks objectivity and reproducibility. MRI (especially non-EP diffusion-weighted imaging) can be used to identify some of them [5–7], but there are multiple limitations, including large regional differences in the number of facilities that can perform MRI studies [8], low temporal resolution, high cost, time-consuming and contraindications, such as internal metal and claustrophobia. Furthermore, even if MRI is performed, its limited resolution makes it difficult to accurately determine an extent of the disease (Fig 1B). Therefore, it is important to improve the diagnostic accuracy of CT.

Recent innovations in artificial intelligence (AI) and machine learning technology have provided a foundation for significant advancements in the medical field. While handwritten prediction algorithms have long been used to aid medical decision-making, the practical application of machine learning methods for prediction began in 2000. Subsequently, dramatic improvements in computer hardware performance led to the introduction of deep neural networks (DNNs) in 2010. In 2012, the accuracy of DNNs exceeded that of conventional image processing methods in the ImageNet Large Scale Visual Recognition Challenge, eventually surpassing the accuracy of human image recognition in 2015. However, large amounts of data are typically required to train DNN models, and their application in diagnosing rare diseases remains challenging. Nevertheless, an AI study showed a high accuracy of hysteroscopy with a small number of samples [9]. Therefore, developing system analysis methods that improve the accuracy with such a small number of samples is an important issue for medical AI research.

Although cholesteatoma is one of the most common middle ear diseases for which surgery is performed, its low prevalence (1-in-25,000) further limits the use of DNN training data. Therefore, this study aimed to develop a method that facilitates an accurate diagnosis of the extent of cholesteatoma extension, despite the limited number of cases in the training dataset. We also compared our DNN models with the assessments performed by otolaryngologists in order to determine their practicability. Notably, our study is the first to demonstrate the feasibility of DNN models for the diagnosis of cholesteatoma extension using CT images.

## Materials and methods

### Patient selection

This study protocol was approved by the Human Ethics Review Committee of the Jikei University School of Medicine. The requirement for informed consent was waived because this was a retrospective study.

We retrospectively evaluated 164 consecutive patients (104 men and 60 women; age range, 13–82 years; average age [±SD], 42.0 ± 15.3 years), including 83 cases of right-sided cholesteatoma and 81 cases of left-sided cholesteatoma, who underwent their first surgery for pars flaccida cholesteatoma and temporal bone HRCT for pretreatment evaluation at the Department of Otorhinolaryngology, Jikei University Hospital, Tokyo, Japan between 2011 and 2020. Pars flaccida cholesteatoma was surgically confirmed in all cases. The diagnosis of cholesteatoma was based on the presence of intraoperative keratinized squamous epithelium and middle ear debris and/or histopathological examination of the excised tissue. All cases were sub-classified into two groups: cases showing extension to the mastoid (M+) and those that did not show an extension to the mastoid (M-). A total of 80 and 84 cases were classified as M+ and M-, respectively (Table 1).

**Table 1:** The number of extracted slices.

Intraoperative findings	Patients	Total Images	Slices including lesion
M (-)	84	2520	912
M (+)	80	2430	1513

All cases were sub-classified into two groups: cases showing extension to the mastoid (M+) and those that did not show an extension to the mastoid (M-). A total of 80 and 84 cases were classified as M+ and M −, respectively. CT slices, including the lesion, were extracted for the training and evaluation of the DNN models.

### High-resolution computed tomography

HRCT of the temporal bone without contrast was performed using a 64-MDCT scanner (SOMATOM Perspective; Siemens AG, Munich, Germany). The CT scans were performed in the supine position. The scanning parameters were as follows: collimation, 64 × 0.6 mm; rotation time, 1.0 s; detector-row width, 0.6 mm; pitch, 1.0; and scanning field of view (FOV), 25 cm. The peak tube voltage is maintained at 130 kV. The reconstruction parameters were as follows: section thickness, 0.6 mm and 0.6-mm reconstruction in the axial plane. The CT threshold was adjusted using the bone algorithm (window center and width were fixed at 700 and 4000, respectively).

Patient information was excluded, and only axial images were used to extract 30 slices caudally from the upper end of the superior semicircular canal cholesteatoma confirmed to be present in this region.

### Datasets

CT slices, including the lesion, were extracted for the training and evaluation of the DNN models. Table 1 lists the number of slices that were extracted. For cross-validation, we randomly divided the patients into eight groups and prepared eight datasets, using seven groups for training and the remaining group for evaluation. Each group was designed such that the number of patients and images were as uniform as possible. Table 2 shows the number of patients and the images for each group.

**Table 2:** The number of patients and images in each group.

Group	M (-)		M (+)	
	Patients	Images	Patients	Images
A	11	116	10	190
B	10	113	10	190
C	10	113	10	190
D	10	113	10	189
E	11	115	10	190
F	11	115	10	188
G	11	115	10	190
H	10	112	10	186

For cross-validation, we randomly divided the patients into eight groups and prepared eight datasets, using seven groups for training and the remaining group for evaluation.

### Neural network and training

In this study, we adopted the MobileNet-V2 network, which is a relatively compact network consisting of 88 layers, with a fixed input image size of 224 × 244 and 3,538,984 learning parameters. The original images were augmented 40 times for both the early and late stages in one epoch. Augmentation was performed randomly without considering the balance between the number of original CT slices for each patient. During training, the DNN models were trained using images cropped to a size of 224 × 224 pixels, keeping the lesion area of the image within the scope. In one training cycle, 50 epochs were repeatedly performed to train one DNN model. This 50-epoch training procedure was performed using eight datasets, and eight models were generated using one training set (training set: evaluation set = 7:1). Because DNN models exhibit differences in ability each time they are trained using a large amount of data generated via augmentation from a small number of patients, 24 training sets were created to verify the accuracy fluctuations of each model. Consequently, 192 models were generated (eight datasets × 24 = 192 models). Table 3 shows the number of patients and the original images in each training set.

**Table 3:** The number of patients and original images in each training set.

Training Set	Training					Evaluation				
	Group	M (-)		M (+)		Group	M (-)		M (+)	
		Patients	Images	Patients	Images		Patients	Images	Patients	Images
Set-1	A,B,C,D,E,F,G	74	800	70	1327	H	10	112	10	186
Set-2	B,C,D,E,F,G,H	73	796	70	1323	A	11	116	10	190
Set-3	C,D,E,F,G,H,A	74	799	70	1323	B	10	113	10	190
Set-4	D,E,F,G,H,A,B	74	799	70	1323	C	10	113	10	190
Set-5	E,F,G,H,A,B,C	74	799	70	1324	D	10	113	10	189
Set-6	F,G,H,A,B,C,D	73	797	70	1323	E	11	115	10	190
Set-7	G,H,A,B,C,D,E	73	797	70	1325	F	11	115	10	188
Set-8	H,A,B,C,D,E,F	73	797	70	1323	G	11	115	10	190

We created 24 training sets to verify the accuracy fluctuations of each model. Consequently, 192 models were generated (8 datasets × 24 = 192 models).

### Evaluation

**Fig 2:**
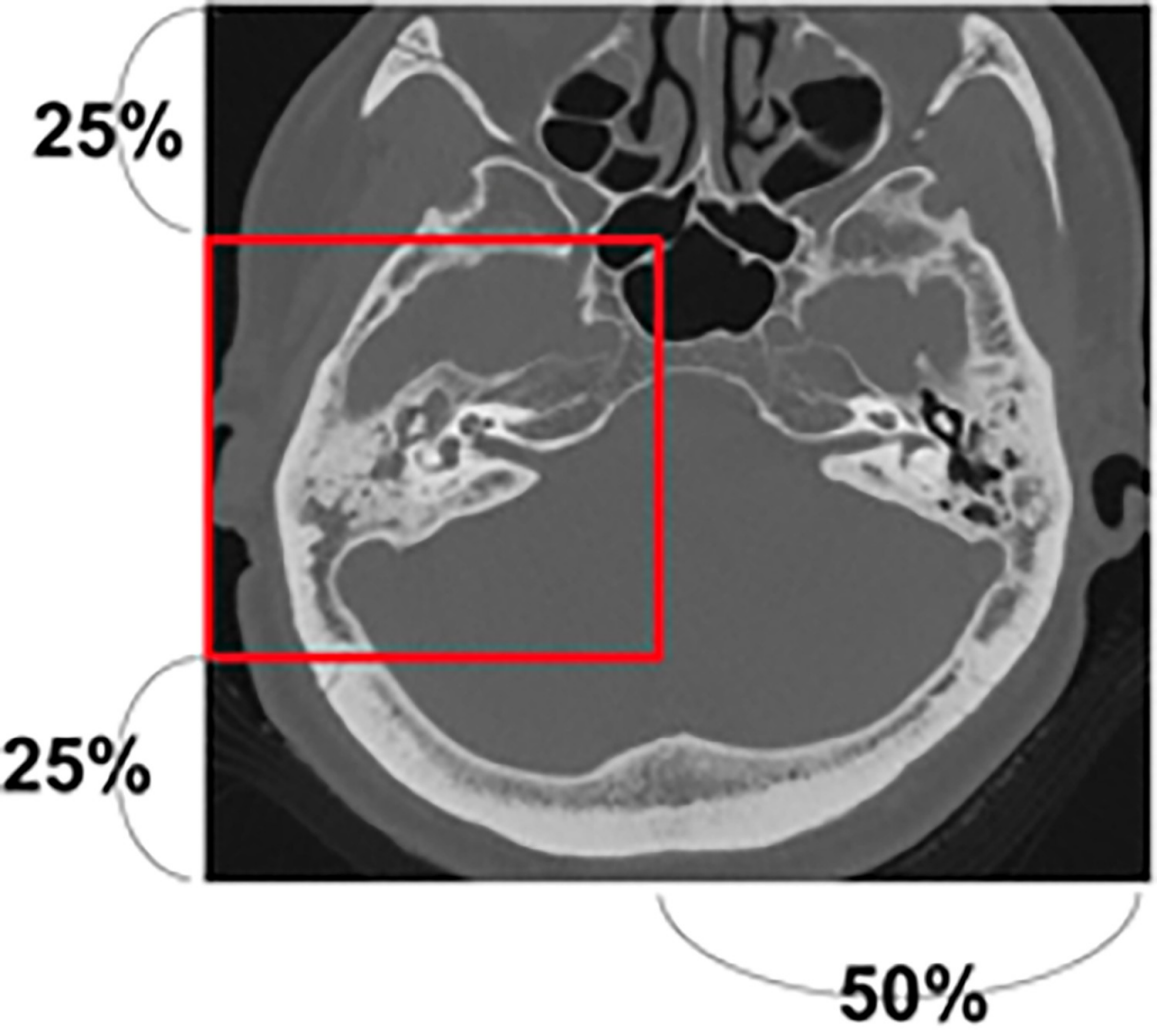
CT images used. We evaluated the accuracy of prediction using 25% partial CT images that contain lesion areas cropped from the vertical center 50% and horizontally left or right 50%.

We used squared CT images that were resized to 224 × 224 pixels. The eight models obtained in each training set were used as a single evaluation set, and the predictions for the 24 evaluation sets were performed as single-image-unit-based-prediction and patient-unit-based-prediction. The single-image-unit-based prediction was performed on every single image, and the patient-unit-based prediction was performed on sets of all images belonging to each patient. In addition to single-model predictions, 24 sets of ensemble predictions combining 23 of the 24 models were used to evaluate the accuracy of the image unit-based prediction and patient unit-based prediction methods. Furthermore, we evaluated the accuracy of the prediction using 25% partial CT images that contained lesion areas cropped from the vertical center 50% and horizontally left or right 50% (Fig 2).

### Evaluation of CT scans by otolaryngologists

Fifteen otolaryngologists with 3–33 years of experience independently and blindly diagnosed patients with or without mastoid extension by looking at the same images that the AI evaluated. The accuracy with which the presence or absence of intraoperative mastoid extension could be correctly predicted by axial CT image alone was evaluated, and the differences in years of experience are shown in the scatterplot.

## Results

### Single-image unit-based prediction / patient unit-based prediction

Tables 4, S1 and S2 shows the sensitivity, specificity, and average of the sensitivity and specificity in single-image unit-based predictions and patient unit-based predictions. The best performance in single-image unit-based predictions was 75.43 on average (sensitivity = 77.12%, specificity = 73.75%), performed by ensemble prediction on 25% cropped images. The best performance in patient unit-based predictions was 81.14% on average (sensitivity = 84.95%, specificity = 77.33%) performed by ensemble prediction on 25% of cropped images. Each number is an average of 24 single models or 24 ensemble predictions performed by the 23 models. This result reveals that ensemble predictions perform better than single model predictions, and prediction on 25% of cropped images yields better performance than the prediction of the original sizes.

**Table 4:** Average accuracy of 24 single models and 24 ensemble predictions in single-image unit-based prediction and patient unit-based prediction.

	Images	Model	Sensitivity	Specificity	Accuracy
Single-Image Unit-Based Prediction	100%	Single	71.87%	62.45%	67.16%
		Ensemble	74.90%	61.76%	68.33%
	25%	Single	80.17%	68.70%	74.43%
		Ensemble	77.12%	73.75%	75.43%
Patient Unit-Based Prediction	100%	Single	80.68%	62.80%	71.74%
		Ensemble	75.31%	74.65%	74.98%
	25%	Single	86.82%	71.97%	79.40%
		Ensemble	84.95%	77.33%	81.14%

The best performance in patient unit-based predictions was 81.14% on average (sensitivity = 84.95%, specificity = 77.33%) performed by ensemble prediction on 25% of cropped images.

**Fig 3:**
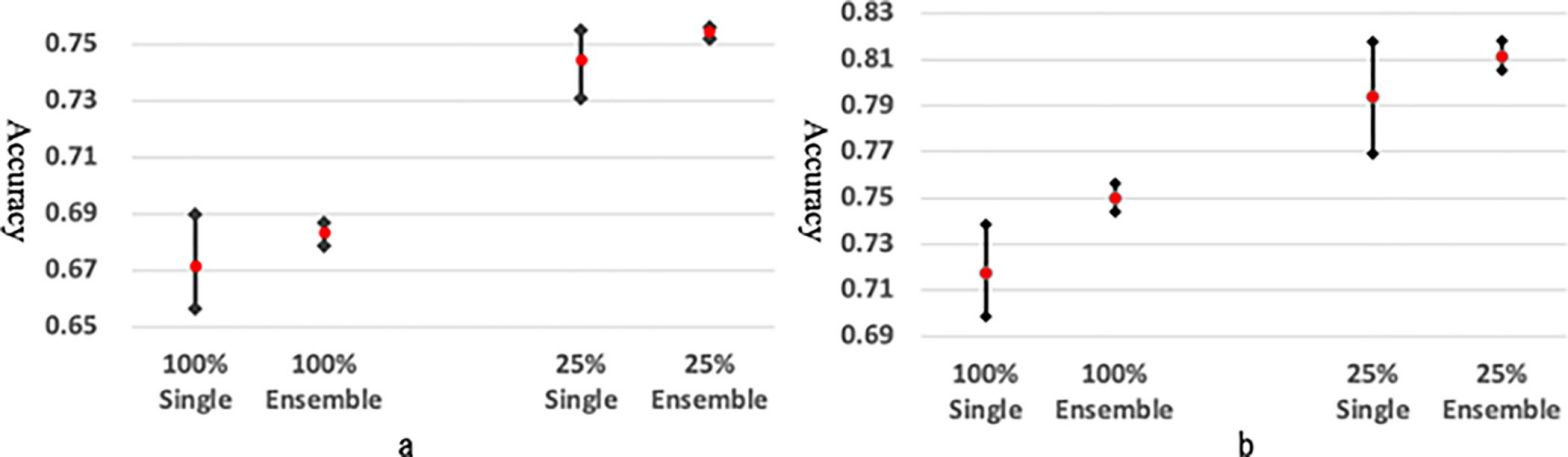
Fluctuation of average accuracy in single-image-unit-based-prediction (a) and patient-unit-based-prediction (b). This chart indicates that the accuracy of prediction on 25% cropped images has a big advantage compared to 100% original size images, and the ensemble predictions perform better than the single model predictions.

The chart in Fig 3 shows the fluctuation in the average accuracy in the single-image unit-based prediction (Fig 3A) and the patient unit-based prediction (Fig 3B). This result reveals that there are large fluctuations in the accuracy of the predictions performed by single models. However, these fluctuations can be reduced by ensemble predictions. This chart also indicates that the accuracy of prediction on 25% cropped images has a significant advantage compared to 100% original size images, and the ensemble predictions result in better performance compared to single model predictions, in both of single-image unit-based prediction and the patient unit-based prediction.

**Fig 4:**
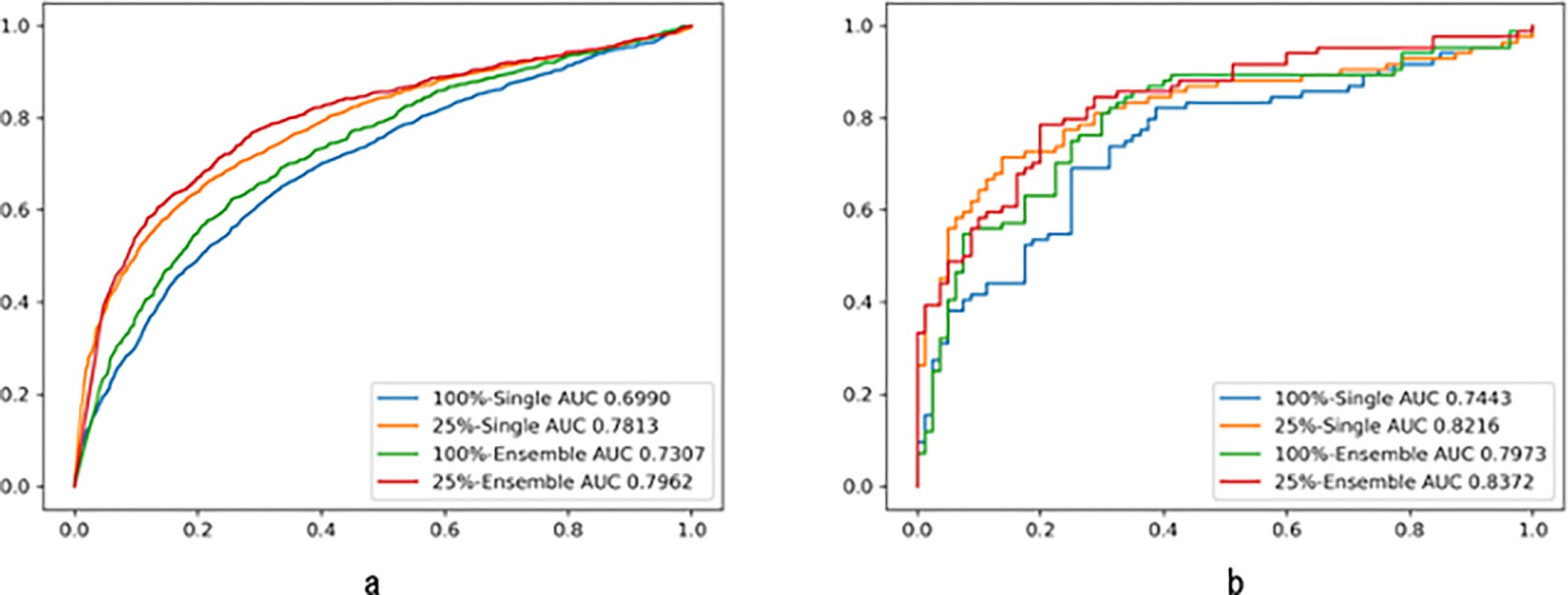
ROC curve of median case in single-image-unit-based-prediction (a) and patient-unit-based-prediction (b). This chart indicates that the best AUC is 0.8372 performed by ensemble predictions on 25% cropped images in the patient unit based prediction. The result reveals predictions on 25% cropped images bring much better performance than 100% original size images regardless of where the thresholds are placed.

The chart in Fig 4 shows the ROC curve of the median case in 24 single model predictions and 24 ensemble predictions in the single image unit based prediction (Fig 4A) and the patient unit-based prediction (Fig 4B). The best AUC is 0.7962 performed by ensemble predictions on 25% cropped images in the single image unit based prediction. The best AUC is 0.8372 performed by ensemble predictions on 25% cropped images in the patient unit based prediction. The result reveals predictions on 25% cropped images bring much better performance than 100% original size images regardless of where the thresholds are placed.

### Diagnostic accuracy by otolaryngologist

**Fig 5:**
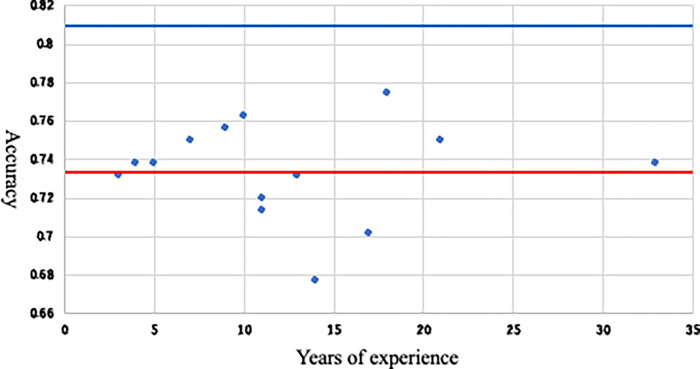
The differences in years of experience are shown in the scatterplot. The red line indicates the average accuracy of the otolaryngologist (73.4%), and the blue line indicates AI accuracy (81.1%).

The average accuracy of otolaryngologists was 73.41% (sensitivity = 83.17%, specificity = 64.13%) and was not affected by their clinical experiences (Fig 5), although this could not be determined definitely because of the small number of otolaryngologists.

## Discussion

CT diagnosis of middle ear cholesteatoma includes the qualitative evaluation and assessment of the extent of the disease. A qualitative diagnosis of middle ear cholesteatomas can generally be made based on clinical findings, such as those obtained in tympanic examinations. However, the diagnosis can be further confirmed based on HRCT findings, such as demineralization of the ossicles (especially the malleus head, incus body, and short crus) or scutum erosion [4]. To evaluate the extent of middle ear cholesteatoma, temporal bone HRCT is useful for preoperative evaluation of cholesteatoma extension, erosion of the ossicles, status of the facial nerve canal, height and erosion of the tegmen tympani, cochlear and semicircular canal fistula, and aeration and development of mastoid cells. In particular, with respect to the extension to the mastoid, the findings of preoperative imaging influence the various decision, including whether to perform a mastoidectomy as well as the indications for transcanal endoscopic ear surgery [10].

In the current study, the average accuracies of the best AI model were 81.14% (sensitivity = 84.95%, specificity = 77.33%) and 73.41% (sensitivity = 83.17%, specificity = 64.13%), respectively. In previous reports on CT diagnosis of mastoid extension, Badran et al. reported a sensitivity of 59% and specificity of 80% for mastoid extension in middle ear cholesteatoma [11] and Razek et al. reported a sensitivity of 79% for mastoid extension [12]. However, in their study, the mastoid sinus extension was visually determined, leading to the possibility that the results could vary depending on the evaluator’s ability and experience levels. Baba et al. reported a qualitative method of diagnosis by measuring the distance in the anterior region of the mastoid sinus (the cut-off value was 3.6 mm [sensitivity, 0.71; specificity, 0.84]) [13]. However, detailed measurements are not easy or precise, and they also take time compared to AI-based diagnosis.

**Fig 6:**
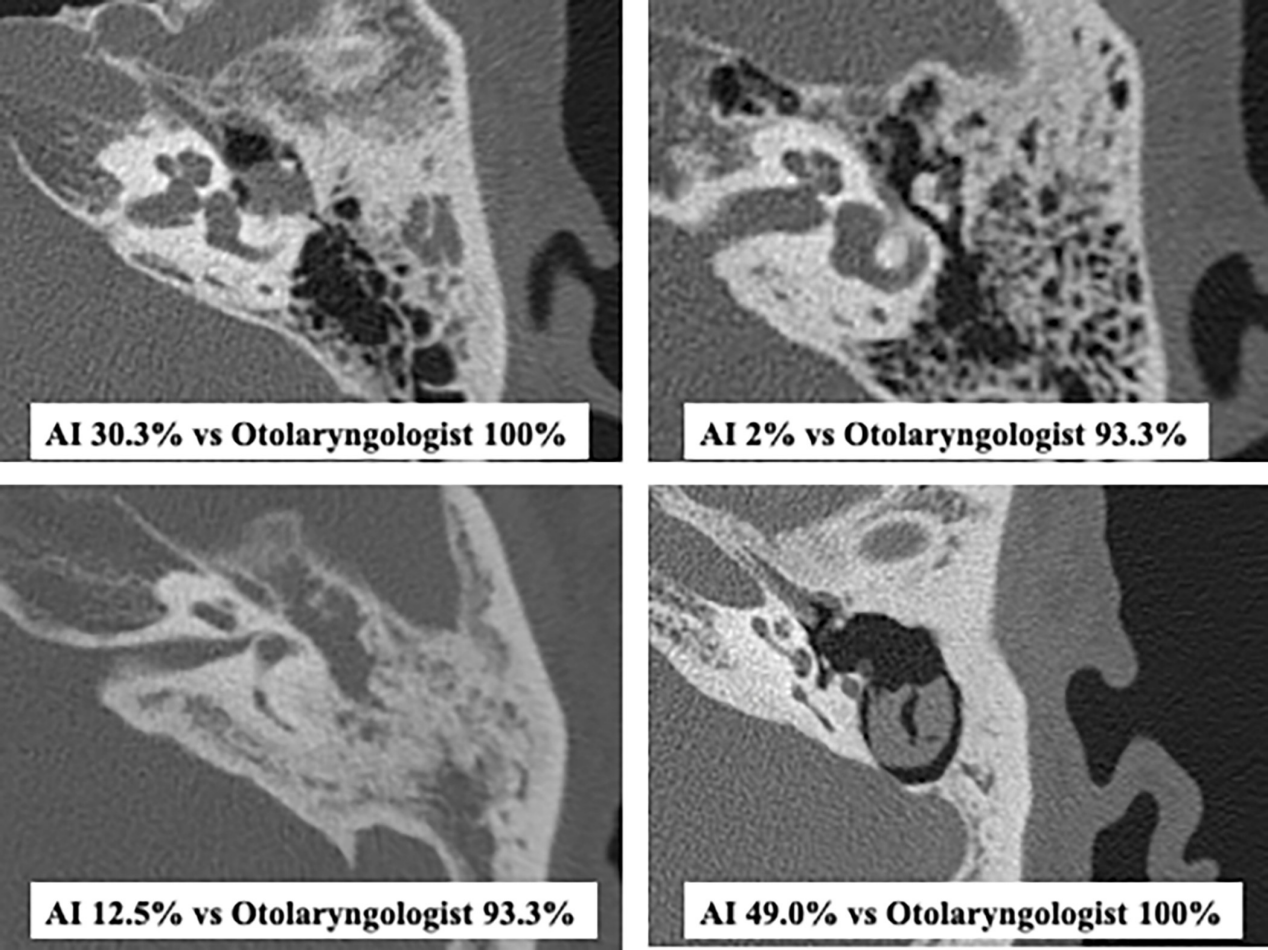
Cases easily diagnosed by otolaryngologists but with low AI accuracy. Upper: Case without mastoid extension. Lower: Case with mastoid extension.

Although the accuracy of our AI model was relatively high, to further improve the diagnostic accuracy, we examined the difference between AI and human diagnosis based on intraoperative findings and CT densities. The results showed that otolaryngologists had a 15–20% difference in the presence or absence of intraoperative mastoid cavity extension and the presence or absence of CT density, whereas AI had a difference of less than 10% (Table 5). Furthermore, the difference based on whether the intraoperative findings matched the density on CT was 65.1% (87.7–22.6%) for otolaryngologists versus 33.2% (84.0–50.8%) for AI (Table 5). This indicates that otolaryngologists judge mainly by the presence or absence of densities, while AI does not, and that AI has a higher percentage of correct responses in cases that are difficult for humans to judge. However, some cases were easily diagnosed by otolaryngologists but had low AI accuracy (Fig 6). We believe that this is because otolaryngologists can distinctively define the mastoid cavity and that even common findings for otolaryngologists in cases of marked extension are insufficient for AI to perform well when the images are new to them [14]. Therefore, increasing the number of cases is desirable.

**Table 5:** Differences between AI and humans in intraoperative findings and CT densities.

	Intraoperative mastoid extension		Mastoid density on CT		Agreement between intraoperative findings and mastoid density on CT	
	No (n = 84)	Yes (n = 80)	No (n = 58)	Yes (n = 106)	No (n = 36)	Yes (n = 128)
Accuracy of otolaryngologists	64.2%	83.1%	83.3%	68.0%	22.6%	87.7%
Accuracy of AI	71.7%	82.0%	80.8%	74.5%	50.8%	84.0%

Otolaryngologists had a 15–20% difference in the presence or absence of intraoperative mastoid cavity extension and the presence or absence of CT density, whereas AI had a less than 10% difference. The difference based on whether the intraoperative findings matched the density on CT was 65.1% (87.7–22.6%) for otolaryngologists vs. 33.2% (84.0–50.8%) for AI.

Despite these findings, our study had several limitations. This was a single-center retrospective study with a small number of patients and included only findings of mastoid extension. Therefore, larger multicenter studies are required. Nevertheless, the fact that this study was able to obtain reasonable results with a small number of cases sheds light on the future of research on rare diseases. This is because the most time-consuming and labor-intensive part of AI R&D in diagnostic imaging is the data collection and processing of the data needed to create AI algorithms. The key is to analyze a small number of cases, and the only way to do this is to accumulate data one by one.

## Conclusion

We examined the mastoid extension of cholesteatoma on axial CT using AI. It is noteworthy that, despite the small number of cases, we were able to create a highly accurate AI. These findings represent an important first step in the automatic diagnosis of cholesteatoma extension.

## Supporting information

S1 Table Single image unit based details. (DOCX)

S2 Table Patient unit based details. (DOCX)
