# Supplementary material for: Preoperative prediction by artificial intelligence for mastoid extension in pars flaccida cholesteatoma using temporal bone high-resolution computed tomography: A retrospective study
Source: PLoS One. 2022 Oct 3;17(10):e0273915. doi: 10.1371/journal.pone.0273915 (PMC9529134; doi:10.1371/journal.pone.0273915)
Supplement: S1 Table — (DOCX) [file pone.0273915.s001.docx]

# Supplementary Table 1

## **Single Image Unit Based Details**

| **Image size** | **Ensemble** | **Set No** | **AUC** | **Threshold** | **Sensitivity** | **Specificity** | **Average** |
| --- | --- | --- | --- | --- | --- | --- | --- |
| 100% | Single | 1 | 0.7083 | 0.6440 | 0.6458 | 0.6973 | 0.6716 |
| 100% | Single | 2 | 0.7077 | 0.5277 | 0.6075 | 0.7297 | 0.6686 |
| 100% | Single | 3 | 0.7282 | 0.7564 | 0.5954 | 0.7845 | 0.6900 |
| 100% | Single | 4 | 0.7069 | 0.8376 | 0.5768 | 0.7581 | 0.6674 |
| 100% | Single | 5 | 0.7098 | 0.5847 | 0.6118 | 0.7132 | 0.6625 |
| 100% | Single | 6 | 0.7131 | 0.0797 | 0.6985 | 0.6345 | 0.6665 |
| 100% | Single | 7 | 0.7087 | 0.7276 | 0.5757 | 0.7548 | 0.6652 |
| 100% | Single | 8 | 0.7132 | 0.4464 | 0.6480 | 0.7032 | 0.6756 |
| 100% | Single | 9 | 0.7168 | 0.6070 | 0.6206 | 0.7244 | 0.6725 |
| 100% | Single | 10 | 0.7046 | 0.7886 | 0.5680 | 0.7561 | 0.6620 |
| 100% | Single | 11 | 0.7192 | 0.1335 | 0.6875 | 0.6596 | 0.6736 |
| 100% | Single | 12 | 0.7029 | 0.4015 | 0.6590 | 0.6973 | 0.6781 |
| 100% | Single | 13 | 0.7122 | 0.6580 | 0.6239 | 0.7224 | 0.6732 |
| 100% | Single | 14 | 0.6959 | 0.5847 | 0.6239 | 0.7098 | 0.6669 |
| 100% | Single | 15 | 0.7186 | 0.3359 | 0.6436 | 0.7052 | 0.6744 |
| 100% | Single | 16 | 0.7090 | 0.4486 | 0.6404 | 0.7112 | 0.6758 |
| 100% | Single | 17 | 0.7161 | 0.3732 | 0.6524 | 0.6927 | 0.6725 |
| 100% | Single | 18 | 0.7235 | 0.7488 | 0.6020 | 0.7555 | 0.6787 |
| 100% | Single | 19 | 0.7154 | 0.8888 | 0.5625 | 0.7911 | 0.6768 |
| 100% | Single | 20 | 0.7085 | 0.3752 | 0.6645 | 0.7026 | 0.6835 |
| 100% | Single | 21 | 0.7039 | 0.9425 | 0.5055 | 0.8077 | 0.6566 |
| 100% | Single | 22 | 0.7171 | 0.0899 | 0.7072 | 0.6391 | 0.6732 |
| 100% | Single | 23 | 0.7046 | 0.4861 | 0.6184 | 0.6966 | 0.6575 |
| 100% | Single | 24 | 0.7120 | 0.3517 | 0.6480 | 0.7013 | 0.6746 |
| 100% | Ensemble | 1 | 0.7162 | 0.9995 | 0.6009 | 0.7726 | 0.6868 |
| 100% | Ensemble | 2 | 0.7160 | 0.9990 | 0.6064 | 0.7614 | 0.6839 |
| 100% | Ensemble | 3 | 0.7117 | 0.9983 | 0.6239 | 0.7336 | 0.6788 |
| 100% | Ensemble | 4 | 0.7144 | 0.9995 | 0.5965 | 0.7687 | 0.6826 |
| 100% | Ensemble | 5 | 0.7154 | 0.9995 | 0.5965 | 0.7707 | 0.6836 |
| 100% | Ensemble | 6 | 0.7169 | 0.9996 | 0.5976 | 0.7733 | 0.6854 |
| 100% | Ensemble | 7 | 0.7162 | 0.9995 | 0.5987 | 0.7713 | 0.6850 |
| 100% | Ensemble | 8 | 0.7154 | 0.9995 | 0.5954 | 0.7680 | 0.6817 |
| 100% | Ensemble | 9 | 0.7162 | 0.9995 | 0.5998 | 0.7673 | 0.6836 |
| 100% | Ensemble | 10 | 0.7145 | 0.9990 | 0.6305 | 0.7356 | 0.6831 |
| 100% | Ensemble | 11 | 0.7153 | 0.9982 | 0.6535 | 0.7118 | 0.6827 |
| 100% | Ensemble | 12 | 0.7156 | 0.9990 | 0.6316 | 0.7350 | 0.6833 |
| 100% | Ensemble | 13 | 0.7144 | 0.9982 | 0.6480 | 0.7138 | 0.6809 |
| 100% | Ensemble | 14 | 0.7160 | 0.9984 | 0.6458 | 0.7191 | 0.6825 |
| 100% | Ensemble | 15 | 0.7159 | 0.9995 | 0.5976 | 0.7693 | 0.6835 |
| 100% | Ensemble | 16 | 0.7155 | 0.9995 | 0.5987 | 0.7680 | 0.6833 |
| 100% | Ensemble | 17 | 0.7156 | 0.9995 | 0.5998 | 0.7673 | 0.6836 |
| 100% | Ensemble | 18 | 0.7141 | 0.9995 | 0.5987 | 0.7667 | 0.6827 |
| 100% | Ensemble | 19 | 0.7132 | 0.9995 | 0.5943 | 0.7673 | 0.6808 |
| 100% | Ensemble | 20 | 0.7159 | 0.9982 | 0.6546 | 0.7125 | 0.6835 |
| 100% | Ensemble | 21 | 0.7170 | 0.9982 | 0.6546 | 0.7138 | 0.6842 |
| 100% | Ensemble | 22 | 0.7161 | 0.9995 | 0.5976 | 0.7700 | 0.6838 |
| 100% | Ensemble | 23 | 0.7155 | 0.9982 | 0.6535 | 0.7151 | 0.6843 |
| 100% | Ensemble | 24 | 0.7160 | 0.9985 | 0.6469 | 0.7224 | 0.6847 |
| 25% | Single | 1 | 0.7830 | 0.7761 | 0.6480 | 0.8308 | 0.7394 |
| 25% | Single | 2 | 0.7945 | 0.8313 | 0.6382 | 0.8334 | 0.7358 |
| 25% | Single | 3 | 0.8041 | 0.5637 | 0.6897 | 0.8143 | 0.7520 |
| 25% | Single | 4 | 0.7963 | 0.3059 | 0.7029 | 0.7832 | 0.7430 |
| 25% | Single | 5 | 0.7988 | 0.4030 | 0.6875 | 0.8077 | 0.7476 |
| 25% | Single | 6 | 0.8037 | 0.2707 | 0.7160 | 0.7845 | 0.7503 |
| 25% | Single | 7 | 0.7903 | 0.9066 | 0.6447 | 0.8295 | 0.7371 |
| 25% | Single | 8 | 0.7746 | 0.1770 | 0.6985 | 0.7634 | 0.7309 |
| 25% | Single | 9 | 0.7989 | 0.6217 | 0.6754 | 0.8110 | 0.7432 |
| 25% | Single | 10 | 0.7882 | 0.3852 | 0.7061 | 0.7746 | 0.7404 |
| 25% | Single | 11 | 0.7992 | 0.8310 | 0.6645 | 0.8229 | 0.7437 |
| 25% | Single | 12 | 0.7984 | 0.5036 | 0.6886 | 0.8037 | 0.7461 |
| 25% | Single | 13 | 0.8071 | 0.5522 | 0.6952 | 0.8070 | 0.7511 |
| 25% | Single | 14 | 0.7891 | 0.7825 | 0.6656 | 0.8024 | 0.7340 |
| 25% | Single | 15 | 0.7982 | 0.3006 | 0.7050 | 0.7812 | 0.7431 |
| 25% | Single | 16 | 0.7873 | 0.3854 | 0.7072 | 0.7759 | 0.7416 |
| 25% | Single | 17 | 0.7877 | 0.1127 | 0.7171 | 0.7469 | 0.7320 |
| 25% | Single | 18 | 0.7981 | 0.2895 | 0.7182 | 0.7720 | 0.7451 |
| 25% | Single | 19 | 0.8002 | 0.4523 | 0.7149 | 0.7951 | 0.7550 |
| 25% | Single | 20 | 0.8039 | 0.3064 | 0.7149 | 0.7951 | 0.7550 |
| 25% | Single | 21 | 0.7921 | 0.5854 | 0.6798 | 0.8044 | 0.7421 |
| 25% | Single | 22 | 0.8033 | 0.7965 | 0.6689 | 0.8387 | 0.7538 |
| 25% | Single | 23 | 0.8008 | 0.9399 | 0.6480 | 0.8553 | 0.7516 |
| 25% | Single | 24 | 0.7959 | 0.4887 | 0.6919 | 0.8077 | 0.7498 |
| 25% | Ensemble | 1 | 0.8015 | 0.9976 | 0.7325 | 0.7773 | 0.7549 |
| 25% | Ensemble | 2 | 0.7988 | 0.9894 | 0.7489 | 0.7581 | 0.7535 |
| 25% | Ensemble | 3 | 0.8005 | 0.9965 | 0.7281 | 0.7832 | 0.7556 |
| 25% | Ensemble | 4 | 0.8013 | 0.9967 | 0.7401 | 0.7673 | 0.7537 |
| 25% | Ensemble | 5 | 0.8012 | 0.9975 | 0.7346 | 0.7733 | 0.7540 |
| 25% | Ensemble | 6 | 0.8011 | 0.9950 | 0.7533 | 0.7508 | 0.7521 |
| 25% | Ensemble | 7 | 0.8009 | 0.9975 | 0.7346 | 0.7773 | 0.7560 |
| 25% | Ensemble | 8 | 0.8022 | 0.9975 | 0.7357 | 0.7733 | 0.7545 |
| 25% | Ensemble | 9 | 0.8007 | 0.9975 | 0.7346 | 0.7759 | 0.7553 |
| 25% | Ensemble | 10 | 0.8006 | 0.9972 | 0.7368 | 0.7700 | 0.7534 |
| 25% | Ensemble | 11 | 0.8011 | 0.9967 | 0.7412 | 0.7700 | 0.7556 |
| 25% | Ensemble | 12 | 0.8012 | 0.9967 | 0.7423 | 0.7660 | 0.7542 |
| 25% | Ensemble | 13 | 0.8008 | 0.9967 | 0.7379 | 0.7687 | 0.7533 |
| 25% | Ensemble | 14 | 0.8007 | 0.9969 | 0.7379 | 0.7680 | 0.7530 |
| 25% | Ensemble | 15 | 0.8014 | 0.9967 | 0.7423 | 0.7667 | 0.7545 |
| 25% | Ensemble | 16 | 0.8017 | 0.9975 | 0.7346 | 0.7766 | 0.7556 |
| 25% | Ensemble | 17 | 0.8015 | 0.9975 | 0.7357 | 0.7759 | 0.7558 |
| 25% | Ensemble | 18 | 0.8002 | 0.9976 | 0.7303 | 0.7779 | 0.7541 |
| 25% | Ensemble | 19 | 0.8005 | 0.9967 | 0.7423 | 0.7673 | 0.7548 |
| 25% | Ensemble | 20 | 0.8000 | 0.9975 | 0.7346 | 0.7726 | 0.7536 |
| 25% | Ensemble | 21 | 0.8026 | 0.9976 | 0.7314 | 0.7766 | 0.7540 |
| 25% | Ensemble | 22 | 0.8012 | 0.9975 | 0.7346 | 0.7746 | 0.7546 |
| 25% | Ensemble | 23 | 0.8009 | 0.9975 | 0.7336 | 0.7740 | 0.7538 |
| 25% | Ensemble | 24 | 0.8010 | 0.9967 | 0.7412 | 0.7667 | 0.7540 |
