# Supplementary material for: Preoperative prediction by artificial intelligence for mastoid extension in pars flaccida cholesteatoma using temporal bone high-resolution computed tomography: A retrospective study
Source: PLoS One. 2022 Oct 3;17(10):e0273915. doi: 10.1371/journal.pone.0273915 (PMC9529134; doi:10.1371/journal.pone.0273915)
Supplement: S2 Table — (DOCX) [file pone.0273915.s002.docx]

# Supplementary Table 2

## **Patient Unit Based Details**

| **Image size** | **Ensemble** | **Set No** | **AUC** | **Threshold** | **Sensitivity** | **Specificity** | **Average** |
| --- | --- | --- | --- | --- | --- | --- | --- |
| 100% | Single | 1 | 0.7265 | 0.7479 | 0.5595 | 0.8375 | 0.6985 |
| 100% | Single | 2 | 0.7262 | 0.4411 | 0.7024 | 0.7375 | 0.7199 |
| 100% | Single | 3 | 0.7391 | 0.4956 | 0.7024 | 0.7500 | 0.7262 |
| 100% | Single | 4 | 0.7293 | 0.6832 | 0.5714 | 0.8500 | 0.7107 |
| 100% | Single | 5 | 0.7185 | 0.7459 | 0.5476 | 0.8875 | 0.7176 |
| 100% | Single | 6 | 0.7121 | 0.6784 | 0.5357 | 0.8750 | 0.7054 |
| 100% | Single | 7 | 0.7256 | 0.7598 | 0.5000 | 0.9125 | 0.7063 |
| 100% | Single | 8 | 0.7350 | 0.6960 | 0.5833 | 0.8500 | 0.7167 |
| 100% | Single | 9 | 0.7347 | 0.4994 | 0.7024 | 0.7750 | 0.7387 |
| 100% | Single | 10 | 0.7153 | 0.5888 | 0.6429 | 0.7625 | 0.7027 |
| 100% | Single | 11 | 0.7335 | 0.5612 | 0.6548 | 0.8000 | 0.7274 |
| 100% | Single | 12 | 0.7269 | 0.5910 | 0.6429 | 0.7875 | 0.7152 |
| 100% | Single | 13 | 0.7381 | 0.8470 | 0.4762 | 0.9250 | 0.7006 |
| 100% | Single | 14 | 0.7122 | 0.4522 | 0.7143 | 0.7125 | 0.7134 |
| 100% | Single | 15 | 0.7313 | 0.7005 | 0.5833 | 0.8750 | 0.7292 |
| 100% | Single | 16 | 0.7344 | 0.5532 | 0.6548 | 0.8000 | 0.7274 |
| 100% | Single | 17 | 0.7342 | 0.5427 | 0.7024 | 0.7500 | 0.7262 |
| 100% | Single | 18 | 0.7527 | 0.4757 | 0.7262 | 0.7375 | 0.7318 |
| 100% | Single | 19 | 0.7268 | 0.6715 | 0.5714 | 0.8625 | 0.7170 |
| 100% | Single | 20 | 0.7272 | 0.4108 | 0.7262 | 0.7000 | 0.7131 |
| 100% | Single | 21 | 0.7168 | 0.4677 | 0.7024 | 0.7250 | 0.7137 |
| 100% | Single | 22 | 0.7421 | 0.6558 | 0.5952 | 0.8625 | 0.7289 |
| 100% | Single | 23 | 0.7193 | 0.5060 | 0.6905 | 0.7375 | 0.7140 |
| 100% | Single | 24 | 0.7179 | 0.7044 | 0.5833 | 0.8500 | 0.7167 |
| 100% | Ensemble | 1 | 0.7579 | 0.8762 | 0.7500 | 0.7500 | 0.7500 |
| 100% | Ensemble | 2 | 0.7528 | 0.8360 | 0.7500 | 0.7375 | 0.7438 |
| 100% | Ensemble | 3 | 0.7548 | 0.8702 | 0.7381 | 0.7750 | 0.7565 |
| 100% | Ensemble | 4 | 0.7561 | 0.8671 | 0.7500 | 0.7500 | 0.7500 |
| 100% | Ensemble | 5 | 0.7561 | 0.9124 | 0.7143 | 0.7750 | 0.7446 |
| 100% | Ensemble | 6 | 0.7601 | 0.8762 | 0.7500 | 0.7500 | 0.7500 |
| 100% | Ensemble | 7 | 0.7579 | 0.8762 | 0.7500 | 0.7500 | 0.7500 |
| 100% | Ensemble | 8 | 0.7567 | 0.8707 | 0.7500 | 0.7500 | 0.7500 |
| 100% | Ensemble | 9 | 0.7580 | 0.8762 | 0.7500 | 0.7500 | 0.7500 |
| 100% | Ensemble | 10 | 0.7579 | 0.8646 | 0.7500 | 0.7500 | 0.7500 |
| 100% | Ensemble | 11 | 0.7579 | 0.8762 | 0.7500 | 0.7500 | 0.7500 |
| 100% | Ensemble | 12 | 0.7589 | 0.8663 | 0.7381 | 0.7500 | 0.7440 |
| 100% | Ensemble | 13 | 0.7542 | 0.8680 | 0.7500 | 0.7500 | 0.7500 |
| 100% | Ensemble | 14 | 0.7588 | 0.8527 | 0.7619 | 0.7375 | 0.7497 |
| 100% | Ensemble | 15 | 0.7598 | 0.8762 | 0.7500 | 0.7500 | 0.7500 |
| 100% | Ensemble | 16 | 0.7567 | 0.8762 | 0.7500 | 0.7500 | 0.7500 |
| 100% | Ensemble | 17 | 0.7583 | 0.8762 | 0.7500 | 0.7500 | 0.7500 |
| 100% | Ensemble | 18 | 0.7563 | 0.8762 | 0.7500 | 0.7500 | 0.7500 |
| 100% | Ensemble | 19 | 0.7570 | 0.8760 | 0.7500 | 0.7500 | 0.7500 |
| 100% | Ensemble | 20 | 0.7585 | 0.8762 | 0.7500 | 0.7625 | 0.7563 |
| 100% | Ensemble | 21 | 0.7597 | 0.8690 | 0.7500 | 0.7500 | 0.7500 |
| 100% | Ensemble | 22 | 0.7582 | 0.8762 | 0.7500 | 0.7625 | 0.7563 |
| 100% | Ensemble | 23 | 0.7565 | 0.9124 | 0.7143 | 0.7750 | 0.7446 |
| 100% | Ensemble | 24 | 0.7585 | 0.8762 | 0.7500 | 0.7500 | 0.7500 |
| 25% | Single | 1 | 0.8143 | 0.6188 | 0.6905 | 0.9000 | 0.7952 |
| 25% | Single | 2 | 0.8083 | 0.5694 | 0.7381 | 0.8250 | 0.7815 |
| 25% | Single | 3 | 0.8304 | 0.6463 | 0.7143 | 0.9000 | 0.8071 |
| 25% | Single | 4 | 0.8243 | 0.6828 | 0.6548 | 0.9250 | 0.7899 |
| 25% | Single | 5 | 0.8295 | 0.6450 | 0.6667 | 0.9125 | 0.7896 |
| 25% | Single | 6 | 0.8332 | 0.6009 | 0.7381 | 0.8875 | 0.8128 |
| 25% | Single | 7 | 0.8156 | 0.4188 | 0.7976 | 0.7500 | 0.7738 |
| 25% | Single | 8 | 0.7929 | 0.6696 | 0.6429 | 0.9000 | 0.7714 |
| 25% | Single | 9 | 0.8344 | 0.6746 | 0.6548 | 0.9375 | 0.7961 |
| 25% | Single | 10 | 0.8131 | 0.6700 | 0.6905 | 0.9000 | 0.7952 |
| 25% | Single | 11 | 0.8213 | 0.5960 | 0.7262 | 0.8500 | 0.7881 |
| 25% | Single | 12 | 0.8278 | 0.5723 | 0.7262 | 0.8625 | 0.7943 |
| 25% | Single | 13 | 0.8350 | 0.4935 | 0.7976 | 0.8375 | 0.8176 |
| 25% | Single | 14 | 0.8232 | 0.4553 | 0.8214 | 0.7500 | 0.7857 |
| 25% | Single | 15 | 0.8207 | 0.5224 | 0.7381 | 0.8500 | 0.7940 |
| 25% | Single | 16 | 0.8225 | 0.6120 | 0.7262 | 0.8625 | 0.7943 |
| 25% | Single | 17 | 0.8115 | 0.5042 | 0.7619 | 0.8125 | 0.7872 |
| 25% | Single | 18 | 0.8198 | 0.6498 | 0.6667 | 0.9125 | 0.7896 |
| 25% | Single | 19 | 0.8414 | 0.6336 | 0.7381 | 0.8875 | 0.8128 |
| 25% | Single | 20 | 0.8490 | 0.5502 | 0.7381 | 0.8750 | 0.8065 |
| 25% | Single | 21 | 0.8152 | 0.4903 | 0.7262 | 0.8125 | 0.7693 |
| 25% | Single | 22 | 0.8186 | 0.7182 | 0.6429 | 0.9375 | 0.7902 |
| 25% | Single | 23 | 0.8250 | 0.5287 | 0.7619 | 0.8500 | 0.8060 |
| 25% | Single | 24 | 0.8278 | 0.6130 | 0.7143 | 0.9000 | 0.8071 |
| 25% | Ensemble | 1 | 0.8417 | 0.8785 | 0.7738 | 0.8500 | 0.8119 |
| 25% | Ensemble | 2 | 0.8363 | 0.8235 | 0.7857 | 0.8250 | 0.8054 |
| 25% | Ensemble | 3 | 0.8378 | 0.8524 | 0.7619 | 0.8625 | 0.8122 |
| 25% | Ensemble | 4 | 0.8411 | 0.8858 | 0.7619 | 0.8625 | 0.8122 |
| 25% | Ensemble | 5 | 0.8421 | 0.8785 | 0.7738 | 0.8500 | 0.8119 |
| 25% | Ensemble | 6 | 0.8408 | 0.8785 | 0.7738 | 0.8500 | 0.8119 |
| 25% | Ensemble | 7 | 0.8405 | 0.8858 | 0.7619 | 0.8500 | 0.8060 |
| 25% | Ensemble | 8 | 0.8417 | 0.8785 | 0.7738 | 0.8500 | 0.8119 |
| 25% | Ensemble | 9 | 0.8388 | 0.8714 | 0.7738 | 0.8500 | 0.8119 |
| 25% | Ensemble | 10 | 0.8408 | 0.8785 | 0.7738 | 0.8500 | 0.8119 |
| 25% | Ensemble | 11 | 0.8414 | 0.8785 | 0.7738 | 0.8500 | 0.8119 |
| 25% | Ensemble | 12 | 0.8409 | 0.8785 | 0.7738 | 0.8500 | 0.8119 |
| 25% | Ensemble | 13 | 0.8374 | 0.8778 | 0.7738 | 0.8500 | 0.8119 |
| 25% | Ensemble | 14 | 0.8403 | 0.8533 | 0.7738 | 0.8375 | 0.8057 |
| 25% | Ensemble | 15 | 0.8414 | 0.8785 | 0.7738 | 0.8500 | 0.8119 |
| 25% | Ensemble | 16 | 0.8424 | 0.8785 | 0.7738 | 0.8500 | 0.8119 |
| 25% | Ensemble | 17 | 0.8409 | 0.8783 | 0.7738 | 0.8500 | 0.8119 |
| 25% | Ensemble | 18 | 0.8396 | 0.8785 | 0.7738 | 0.8500 | 0.8119 |
| 25% | Ensemble | 19 | 0.8376 | 0.8525 | 0.7738 | 0.8375 | 0.8057 |
| 25% | Ensemble | 20 | 0.8402 | 0.8785 | 0.7738 | 0.8500 | 0.8119 |
| 25% | Ensemble | 21 | 0.8423 | 0.8714 | 0.7857 | 0.8500 | 0.8179 |
| 25% | Ensemble | 22 | 0.8402 | 0.8785 | 0.7738 | 0.8500 | 0.8119 |
| 25% | Ensemble | 23 | 0.8408 | 0.8785 | 0.7738 | 0.8625 | 0.8182 |
| 25% | Ensemble | 24 | 0.8417 | 0.8785 | 0.7738 | 0.8500 | 0.8119 |
